# Molecular adsorption and self-diffusion of NO_2_, SO_2_, and their binary mixture in MIL-47(V) material†

**DOI:** 10.1039/d3ra02724d

**Published:** 2023-06-23

**Authors:** Kompichit Seehamart, Wutthikrai Busayaporn, Rungroj Chanajaree

**Affiliations:** a Department of Applied Physics, Faculty of Engineering, Rajamangala University of Technology Isan Khon Kaen Campus Khon Kaen 40000 Thailand; b Synchrotron Light Research Institute (Public Organization) Nakhon Ratchasima 30000 Thailand; c Metallurgy and Materials Science Research Institfute (MMRI), Chulalongkorn University Bangkok 10330 Thailand Rungroj.Ch@chula.ac.th

## Abstract

The loading dependence of self-diffusion coefficients (*D*_s_) of NO_2_, SO_2_, and their equimolar binary mixture in MIL-47(V) have been investigated by using classical molecular dynamics (MD) simulations. The *D*_s_ of NO_2_ are found to be two orders of magnitude greater than SO_2_ at low loadings and temperatures, and its *D*_s_ decreases monotonically with loading. The *D*_s_ of SO_2_ exhibit two diffusion patterns, indicating the specific interaction between the gas molecules and the MIL-47(V) lattice. The maximum activation energy (*E*_a_) in the pure component and in the mixture for SO_2_ are 16.43 and 18.35 kJ mol^−1^, and for NO_2_ are 2.69 and 1.89 kJ mol^−1^, respectively. It is shown that SO_2_ requires more amount of energy than NO_2_ to increase the diffusion rate. The radial distribution functions (RDFs) of gas–gas and gas–lattice indicate that the Oh of MIL-47(V) are preferential adsorption site for both NO_2_ and SO_2_ molecules. However, the presence of the hydrogen bonding (HB) interaction between the O of SO_2_ and the H of MIL-47(V) and also their binding angle (*θ*(OHC)) of 120° with the linkers of lattice indicate a stronger binding interaction between the SO_2_ and the MIL-47(V), but it does not occur with NO_2_. The jump-diffusion of SO_2_ between adsorption sites within the lattice has been confirmed by the 2D density distribution plots. Moreover, the extraordinarily high *S*_diff_ for NO_2_/SO_2_ of 623.4 shows that NO_2_ can diffuse through the MIL-47(V) significantly faster than SO_2_, especially at low loading and temperature.

## Abstract 2

The loading dependence of self-diffusion coefficients (*D*_s_) of NO_2_, SO_2_, and their equimolar binary mixture in MIL-47(V) have been investigated by using classical molecular dynamics (MD) simulations.

## Graphical abstract

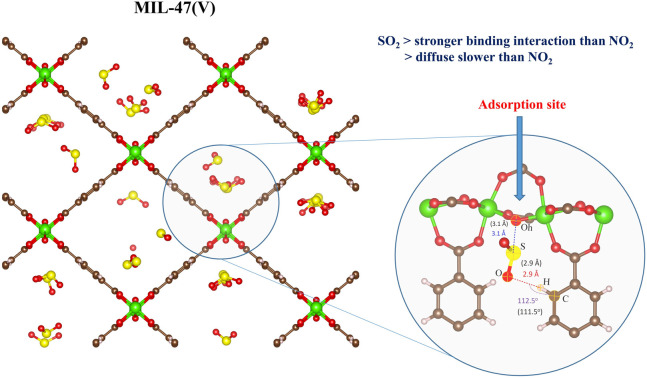

## 1 Introduction

According to the World Health Organization (WHO), one eighth of all worldwide deaths are caused by air pollution.^1,2^ Nitrogen dioxide (NO_2_) and sulfur dioxide (SO_2_) are two of the main air pollutants released during the combustion of fossil fuels such as coal and petroleum.^3–5^ SO_2_ and NO_2_ are constantly present in the atmosphere as a result of the increasing industrialization of several developing countries.^6^ These gases have a high solubility in water and are colorless, non-flammable, and corrosive. Excessive pollution gas emissions will lead to the formation of acid rain, which poses a serious hazard to human health in addition to having a negative influence on the ecosystem.^7^ Therefore, eliminating SO_2_ and NO_2_ from flue gas becomes important in preserving air quality.^4,8^ In case of SO_2_, flue gas desulfurization (FGD) technologies like limestone scrubbing successfully remove a significant portion of 95% of SO_2_ from flue gases, but residual SO_2_ still remains and can harm additional gas scrubbers.^9–12^ Due to the highly reactive and corrosive nature of SO_2_ and NO_2_, complete removal of SO_2_ and NO_2_ traces is difficult and requires a capture system with significant stability and higher selectivity of SO_2_ and NO_2_ than that of N_2_ and CO_2_.^13–16^ Designing new materials is important for removing traces of SO_2_ and NO_2_. In terms of process economy and energy efficiency, it could be a possibility.^17–19^ Metal organic frameworks (MOFs) have showed significant promise in a variety of applications, including drug delivery, substrate binding, and gas adsorption, storage, and separation.^20–23^ Molecular modeling contributions to predict the performance of MOFs play an important role in selecting materials for specific applications. The diffusion behavior of gases is essential in many of the applications proposed for MOFs. Without understanding of gas diffusion rates, the applications for MOFs such as catalysis, membranes, and sensors cannot be assessed. Furthermore, the majority of knowledge on gas diffusion in MOFs has come from molecular dynamics (MD) research.

However, to the best of our knowledge, molecular simulations studies were mostly focused on carbon capture, and much less attention has been given to the removals of SO_2_ and NO_*x*_.^4,24,25^ MOF materials are a promising alternative for the capture of SO_2_ and NO_2_, especially because of their unique chemical functionality and pore sizes, which have shown great results for the capture assignment.^26^ However, the investigations of the adsorption of the strong reactivity molecules *e.g.* SO_2_ and NO_2_, including their mixture in MOFs have rarely been reported.^5,27–29^ Kampouraki *et al*.^30^ reported the removal of SO_2_ by using MOF materials. Their results indicate that the selectivity and adsorption ability are further enhanced after functionalization of the MOF surface. Sun *et al*.^4^ investigated the porous MOFs and zeolites for removal of SO_2_ and NO_*x*_ from flue gases by grand canonical Monte Carlo (GCMC) simulations. They found that Cu-BTC and MIL-47 have high adsorption ability of SO_2_, hence they can be used for the removal of SO_2_ from flue gases. Peng *et al*.^24^ studied many kinds of porous materials. They discovered that the SO_2_ molecules have a very strong binding interaction with the Na-5A and the Na-13X zeolites which is difficult in SO_2_ desorption even at high temperature. While the MIL-47(V) and zeolite-like MOFs (zMOFs) materials are easier to regenerate at high temperature since it has a weaker interaction with SO_2_. López-Olvera *et al*.^31^ reported that the MIL-53(Al)-TDC and the MIL-53(Al)-BDC have excellent SO_2_ adsorption even under humid condition, and also have high chemical stability and easy to regenerate. However, the MIL-53(Al)-TDC which exhibits as a rigid-like lattice has higher SO_2_ adsorption than the MIL-53(Al)-BDC.

**Fig. 1:**
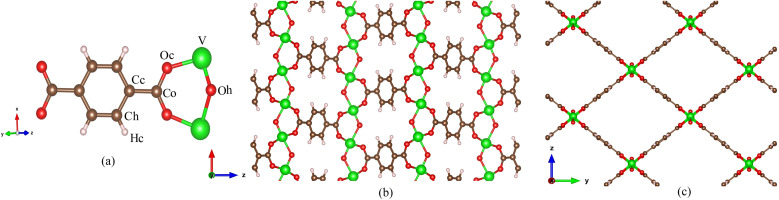
(a) Labeled atom types for MIL-47(V) lattice, (b) MIL-47(V) lattice in *xz* plane and (c) in *yz* plane.

MIL-47(V) is one of MOF materials^32,33^ that was created by Gérard Férey's group.^34^ It is composed of V^4+^O_6_ octahedra linked by linear dicarboxylate ligands, providing a 3D framework with 1D diamond-shaped pores with a diameter of approximately 8.5 Å (see Fig. 1). The material has excellent water stability, a hydrophobic property, a large BET surface area of 930 m^2^ g^−1^, and a high thermal stability in air up to 400 °C.^34–37^ It also shows stability upon adsorption of different adsorbates,^35,38–42^ and its framework does not collapse even under high pressure.^43^

The MIL-47(V) material which acts as a rigid-like lattice similar to the MIL-53(Al)-TDC, has an excellent SO_2_ adsorption and also reported to be an effective H_2_S adsorbent.^41,44,45^ Additionally, it has been brought to the market. However, the diffusion and separation of SO_2_ and NO_2_ in MIL-47(V) material have received limited theoretical and experimental research, despite the fact that numerous research have emphasized on the adsorption in this material.

Using MD simulations, this study has looked into the self-diffusivity of pure SO_2_, NO_2_, and their mixture in a MIL-47(V) lattice at different loadings and temperatures. Radial distribution functions (RDFs) and probability densities were used to analyze the detailed molecular adsorption in MIL-47(V). Our findings were discussed and compared with those from earlier studies.

## 2 Model and simulations

Classical Molecular Dynamics (MD) simulations were conducted to determine the adsorption and self-diffusion coefficient of SO_2_, NO_2_, and their equimolar (1 : 1) mixture in MIL-47(V) material. The MIL-47(V) framework structure is provided from Cambridge Crystallographic Data Center (CCDC-166785).^46^ An orthorhombic unit cell (*Pnma*) of MIL-47(V) contains 72 lattice atoms (C_32_H_16_O_20_V_4_) with lattice parameter of *a* = 6.1879 Å, *b* = 16.1430 Å, and *c* = 13.9390 Å. The simulation box was extended to 32 (8 × 2 × 2) unit cells with dimensions of 54.5432 × 32.2860 × 27.8780 Å, comprise of 2304 lattice atoms (see Fig. 1).

For the SO_2_ and NO_2_ molecules, they were modeled by using the three-site Lennard-Jones model.^4^ The force field parameters for the MIL-47(V) framework were obtained from Xu *et al*.,^47^ which utilizes the DREIDING force field,^48^ except vanadium which utilized the UFF force field.^49^ The Lennard-Jones (LJ) parameters with partial charges which follow eqn (1), are summarized in Table 1.1
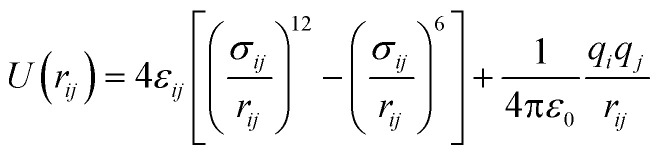
where *ε*_*ij*_ is the well depth, *σ*_*ij*_ is the LJ distance, and *r*_*ij*_ is the distance between two interacting atoms *i* and *j*. The potential parameters between two different species were obtained from the Lorenz-Berthelot mixing rule^50^ written as eqn (2) and (3).

2 *σ*_*ij*_ = (*σ*_*i*_ + *σ*_*j*_)/2

3
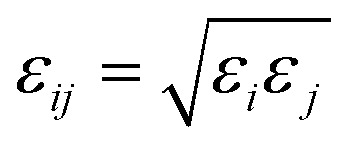


**Table 1:** LJ parameters and atomic partial charges for lattice and guest molecules

Molecules	Atom type	*ε*/*k*_B_ (K)	*σ* (Å)	Charge (e)	Geometry (—)
MIL-47(V)	V	8.056	2.801	0.861	—
	Oh	48.161	3.033	−0.475	—
	Oc	48.161	3.033	−0.446	—
	Co	47.859	3.473	0.603	—
	Ch	47.859	3.473	−0.015	—
	Cc	47.859	3.473	−0.097	—
	Hc	7.649	2.846	0.112	—
SO_2_	S	145.900	3.620	0.471	*d*_S–O_ = 1.43 Å
	O	57.400	3.010	−0.2355	∠OSO = 119.50°
NO_2_	N	50.360	3.240	0.146	*d*_N–O_ = 1.20 Å
	O	62.510	2.930	−0.073	∠OSO = 134.30°

MD simulations of SO_2_, NO_2_, and SO_2_/NO_2_ mixture in the rigid framework of MIL-47(V) were performed under the NVT ensemble at 200, 250, 298, 350, and 400 K with the Nosé-Hoover thermostat.^51^ The periodic boundary condition (PBC) was applied and the shifted potential was truncated with a cutoff distance of 12 Å. The coulombic interactions were described by the Ewald summation with a precision of 10^−5^. The velocity Verlet algorithm was used, with a time step of 1 fs. The equilibration period of 1 ns (1 000 000 steps) and another 5 ns (5 000 000 steps) for sampling density were performed. Then, the production runs were carried out for 15 ns (15 000 000 steps) with trajectory collection taking every 100 fs. The DL_POLY package^52^ has been applied for all simulations. The simulation boxes for SO_2_ and NO_2_ in the MIL-47(V) framework is shown in Fig. S1 in the ESI.†

The self-diffusion coefficients (*D*_s_) of SO_2_ and NO_2_ in pure component and in mixture within the one-dimensional channel of MIL-47(V)^38,53,54^ were computed from the linear fit to the mean-squared displacements (MSDs). It is written as Einstein's relation,^55^ see eqn (4).4
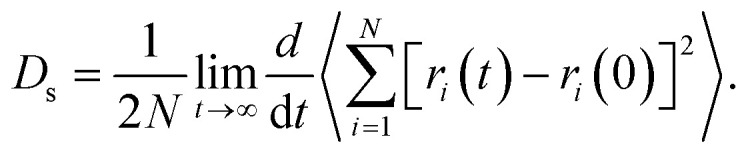
where *r*_*i*_(*t*) is the position of molecule *i* at time *t*, *r*_*i*_(0) is the initial position, and 〈 … 〉 denotes an ensemble average. Note that the MSDs were computed and averaged from five different production runs.

The diffusion selectivities (*S*_diff_)^56^ were defined as the ratio of the *D*_s_ of molecules from species A and B in a binary mixture (eqn (5)).5
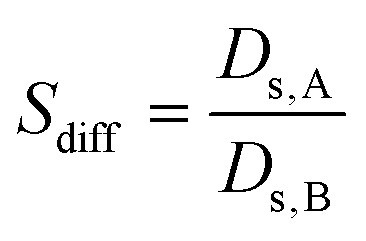
where *D*_s,A_ is the self-diffusion coefficient of molecules of specie A, and *D*_s,B_ is the self-diffusion coefficient of molecules of specie B. When *S*_diff_ is more than 1, it means that specie A can diffuse through the lattice better than specie B.

The activation energies (*E*_a_) for *D*_s_ of SO_2_ and NO_2_ were obtained from the linear slope of the Arrhenius equation which can be written as6
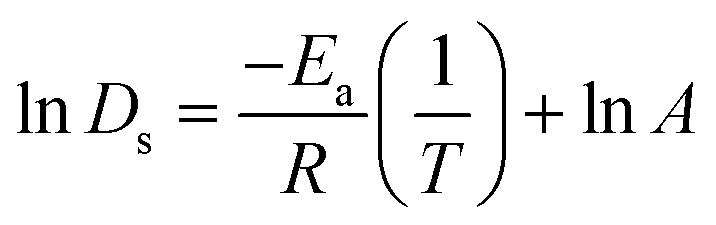
where *R* is the universal gas constant, *T* is the temperature and *A* is the pre-exponential factor.

## 3 Results and discussion

### 3.1 Self-diffusion coefficients (*D*_s_)

**Fig. 2:**
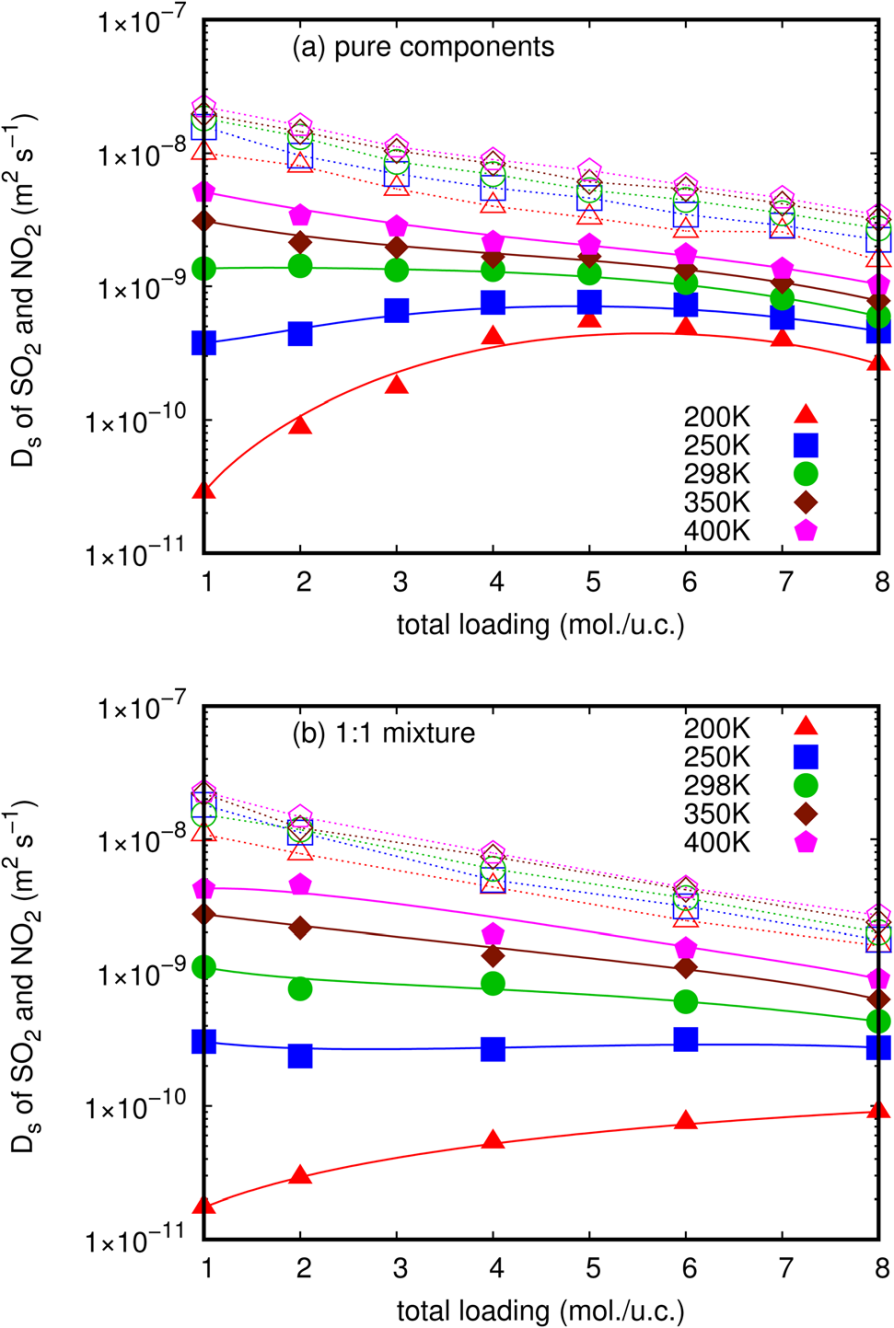
The loading dependence of *D*_s_ of SO_2_ (solid line) and NO_2_ (dashed line) in pure component (a) and in mixture (b) within the MIL-47(V) framework at different temperatures.

In this work, *D*_s_ of NO_2_ and SO_2_ for pure components and their mixture in MIL-47(V) material have been examined using conventional MD simulations as a function of loading at 200, 250, 298, 350, and 400 K. The MSDs in one-dimensional channel of MIL-47(V) were calculated from the center of mass of guest molecules over 5 ns, averaged by shifting the starting point of the evaluation over the first 10 ns of a 15 ns trajectory. The representative MSD plots' approaching linearity shows that the simulation's quality is reliable and that it yields *D*_s_ values (see Figs. S2 and S3†). The MSDs in different axes (*i*.*e*. *x*, *y* and *z*) that indicate the one-dimensional diffusion are illustrated in Fig. S5.†Fig. 2a and b illustrate how temperature and loading affect the *D*_s_ (*i*.*e*. *D*_1*D*/3_) values of NO_2_ and SO_2_ for both their pure components and their mixture, respectively. All *D*_s_ values are also summarized in Tables S1 and S2.†

These figures clearly indicate that NO_2_ molecules in MIL-47(V) have expectedly larger *D*_s_ values than SO_2_ molecules at all loadings and temperatures, as well as in both pure component and mixture. Since the pore diameters of MIL-47(V) (8.5 Å) are greater than the kinetic diameter of NO_2_ (3.3 Å), the diffusion of NO_2_ is governed by steric hindrance between diffusing molecules at higher loading, and the resulting *D*_s_ value decreases monotonically with loading (called type I self-diffusion).^57^ Several guest molecules in MIL-47(V) exhibited similar loading dependent diffusion behavior in both experimental and simulated studies.^35,39,40,42,47^ The *D*_s_ of NO_2_ increase only slightly as temperature increases. In addition, at low loading and temperature, the *D*_s_ of NO_2_ are approximately two orders of magnitude more than those of SO_2_, which has a kinetic diameter of 4.1 Å.

Nevertheless, *D*_s_ of SO_2_ are considerably sensitive to loading and temperature in MIL-47(V) than those of NO_2_, hence the MIL-47(V) material can probably be regenerated at high temperature. *D*_s_ of SO_2_ exhibit two types of self-diffusion behavior, particularly in pure components with loading varied from 1 to 8 mol per u.c. At temperatures over 298 K, its *D*_s_ decreases monotonically as loading increases due to steric hindrance (type I self-diffusion), similar to that of NO_2_. While, at temperatures equal to or less than 298 K, *D*_s_ increases as a function of loading from 1 to 4 mol per u.c., reaches a maximum at 5 mol per u.c., and subsequently decreases with increasing loading (type IV self-diffusion). The diffusing CO_2_ and SO_2_ molecules in ZIF-10,^58^ the CO_2_ molecules in other ZIFs,^37^ and the H_2_S molecules in MIL-47(V) from our previous work revealed similar loading dependent diffusion behavior.^45^

For the mixture in MIL-47(V) at 200 K, the *D*_s_ of SO_2_ increase as total loading increases from 1 to 8 mol per u.c., in which the amount of SO_2_ is 0.5–4 mol per u.c. (see Fig. 2b). This unexpected Type IV evolution of *D*_s_ is most often associated with preferential interactions between the host material and the diffusive species, and the maximum in *D*_s_ is determined by the number of adsorption sites. It is confirmed by the log-log plot of MSD that shows a sub-diffusion process of SO_2_ as shown in Fig. S4.† As a result, the first SO_2_ molecules will stick to stronger adsorption sites and diffuse more slowly than when other molecules are introduced. In addition, the loading dependence for diffusion behavior of SO_2_ molecules differs greatly from that of NO_2_ molecules, which might be attributed to differences in interactions between gas molecules and MIL-47(V). Moreover, the relationship between *D*_s_ and temperature can be described using an Arrhenius equation. The activation energy represents the energy barrier that particles need to overcome to diffuse, and it determines the sensitivity of diffusion to temperature changes. A higher activation energy implies a stronger temperature dependence of diffusion.

### 3.2 Activation energy (*E*_a_)

**Fig. 3:**
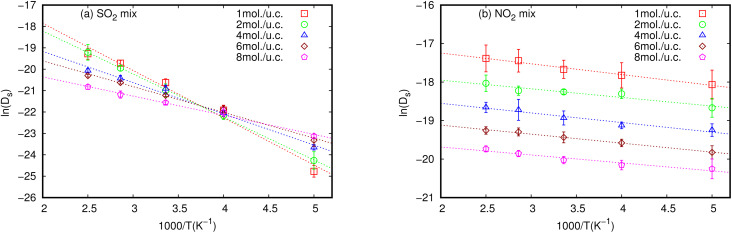
Inverse temperature dependence of the simulated *D*_s_ of (a) SO_2_ and (b) NO_2_ in equimolar mixture in MIL-47(V) at different loadings. The dashed line represent the linear fit for an Arrhenius relationship.

Activation energy (*E*_a_) is an important factor that influences gas diffusion. The activation energy is the least amount of energy required for gas molecules to diffuse from one site to another within the MOF framework, and it is a critical parameter in determining the diffusion rate of gas in the MOF. *E*_a_ is affected by the temperature, the size and shape of the MOF pores or channel, and that the gas molecules interact with the MOF framework. Fig. 3 shows the relationship between *D*_s_ at various loadings that vary with the inverse of temperature.

**Fig. 4:**
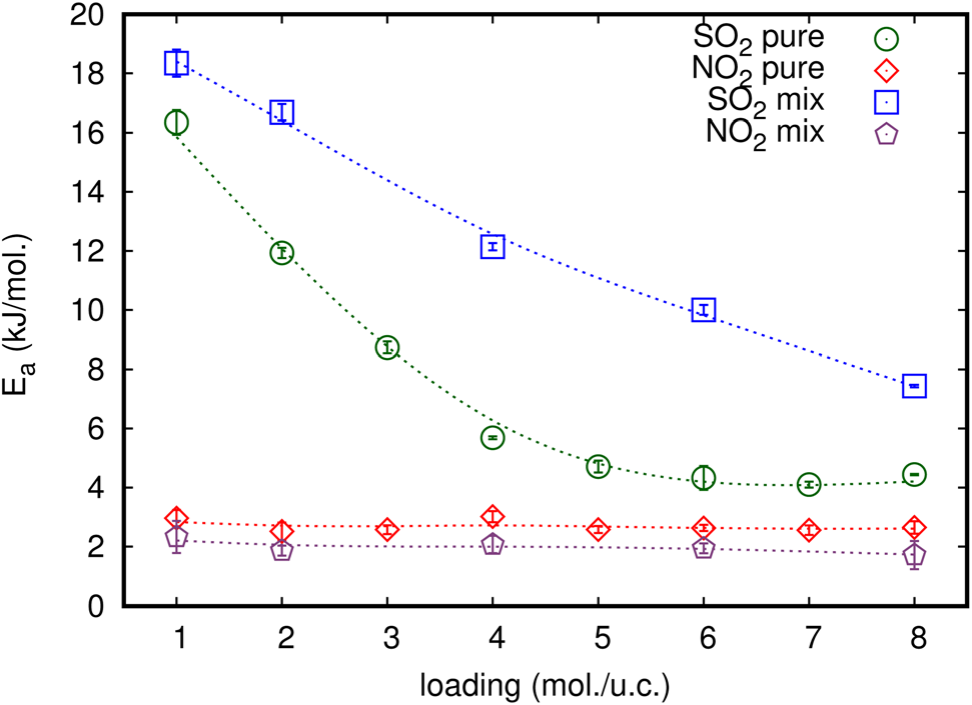
The loading dependence of *E*_a_ of SO_2_ and NO_2_ for pure and their mixture in the MIL-47(V) framework.

The relationship between ln(*D*_s_) and 1/*T* appeared to be linear for all loadings. The *E*_a_ of diffusion of SO_2_ and NO_2_ in MIL-47(V) for pure component and mixture is obtained by fitting ln(*D*_s_) *versus* 1/*T* to the Arrhenius equation (eqn (6)), see Fig. S6 in the ESI† for pure component. The calculated *E*_a_ values is shown in Fig. 4. The *E*_a_ of SO_2_ in the mixture is greater than that in the pure component, and it decreases with increasing loadings for both the mixture and the pure component. For a total loading of 1 mol per u.c., the maximum *E*_a_ values of SO_2_ are 16.34 and 18.35 kJ mol^−1^ for the pure component and the mixture, respectively, suggesting that SO_2_ diffusion in the mixture is slower than that in the pure component. In case of pure component, as the loading varies from 1 to 4 mol per u.c., the *E*_a_ of SO_2_ decrease rapidly, and then become relatively constant when the loading exceeds 5 mol per u.c. This corresponds to Type IV diffusion behavior of SO_2_, as seen in Fig. 2. In the event of a mixture, the *E*_a_ values of SO_2_ remain significantly decreased since the loading of SO_2_ molecules in mixture only varies from 0.5–4 mol per u.c., in which the total loading varies between 1 and 8 mol per uc. It is seen that the activation energy of SO_2_ decreases with increasing loading, due to the repulsive interactions between diffusing molecules which are expected to reduce significantly in SO_2_–MIL-47(V) framework interactions. This increases the diffusion rate of SO_2_ molecules.

On the other hand, the *E*_a_ of NO_2_ in pure component is unaffected by loading and that in mixture is slightly lower than in pure component. The averaged *E*_a_ values of NO_2_ for the pure component and the mixture are 2.69 and 1.89 kJ mol^−1^, respectively. The absence of considerable variability in the *E*_a_ of NO_2_ indicates the existence of uniform NO_2_–NO_2_ and NO_2_–MIL-47(V) interactions.

### 3.3 Adsorption properties

#### 3.3.1 Radial distribution functions (RDFs)

**Fig. 5:**
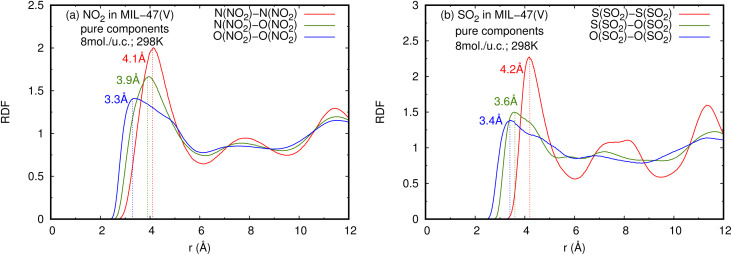
RDFS of (a) NO_2_–NO_2_ and (b) SO_2_–SO_2_ in pure component, at loading of 8 mol per u.c. and 298 K.

**Fig. 6:**
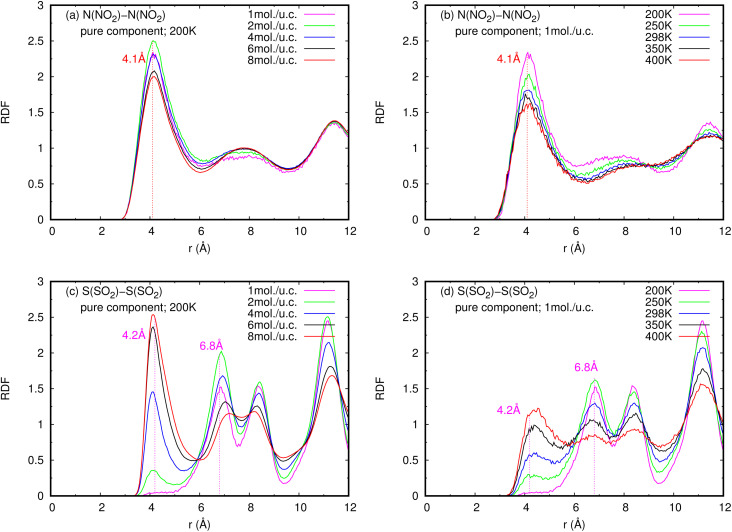
RDFS of (a), (b) NO_2_–NO_2_ and (c), (d) SO_2_–SO_2_ in pure component, at different loadings and temperatures.

The radial distribution functions (RDFs) of NO_2_ and SO_2_ diffusing in the MIL-47(V) framework were calculated from the trajectories stored throughout the MD runs to better understand the microscopic properties at the molecular scale. They are used to determine the typical interparticle distance of NO_2_ and SO_2_ molecules diffusing in MIL-47(V). Fig. 5 displays the RDFs for the pure component gas phase like molecule interactions of NO_2_ and SO_2_ in MIL-47(V) at 298 K. The first peak of N(NO_2_)–N(NO_2_), N(NO_2_)–O(NO_2_), and O(NO_2_)–O(NO_2_) is at 4.1 Å, 3.9 Å, and 3.3 Å, respectively. Similarly, the initial peaks of S(SO_2_)–S(SO_2_), S(SO_2_)–O(SO_2_), and O(SO_2_)–O(SO_2_) are located at 4.1 Å, 3.6 Å, and 3.4 Å, respectively, and they agree very well with the results reported in ref. 59–61. As a result, it can be deduced that the force field used in this research is valid. In order to generate a molecular level picture of the adsorption and diffusion process of NO_2_ and SO_2_ in MIL-47(V) for both pure component and mixture, as well as to obtain more detail of molecular interactions and their positions in the channel of MIL-47(V), the RDFs of N(NO_2_)–N(NO_2_) and S(SO_2_)–S(SO_2_) were further investigated at various loadings and temperatures, as shown in Fig. 6 (for mixture see Fig. S7 in the ESI†). The RDFs of N(NO_2_)–N(NO_2_) do not significantly change as the loading and temperature change, relating to the previously mentioned *D*_s_ tendency of NO_2_. It is seen that the RDFs of N(NO_2_)–N(NO_2_) do not pronounce within 2.2 Å,^62^ shows that there is no dimerization of NO_2_ in the simulations. In contrast to NO_2_, the RDFs of S(SO_2_)–S(SO_2_) are profoundly changed by changes in loading and temperature. At a loading of 1 mol per u.c., the first peak of S(SO_2_)–S(SO_2_) at 4.2 Å, is not distinctly visible. However, this peak is higher at higher loadings and temperatures. Additionally, the second peak, which is visible especially at low loading, is located around 6.8 Å. It is seen that at low loading, SO_2_ molecules are typically 6.8 Å apart from one another. The first peak of S(SO_2_)–S(SO_2_) significantly increases with increasing loading, signifying a stronger SO_2_–SO_2_ interaction. It is also correlated with the increase in the *D*_s_ of SO_2_ at loadings of 1 to 4 mol per u.c. for pure components. Moreover, the RDFs mentioned above are similar to those in mixture component.

**Fig. 7:**
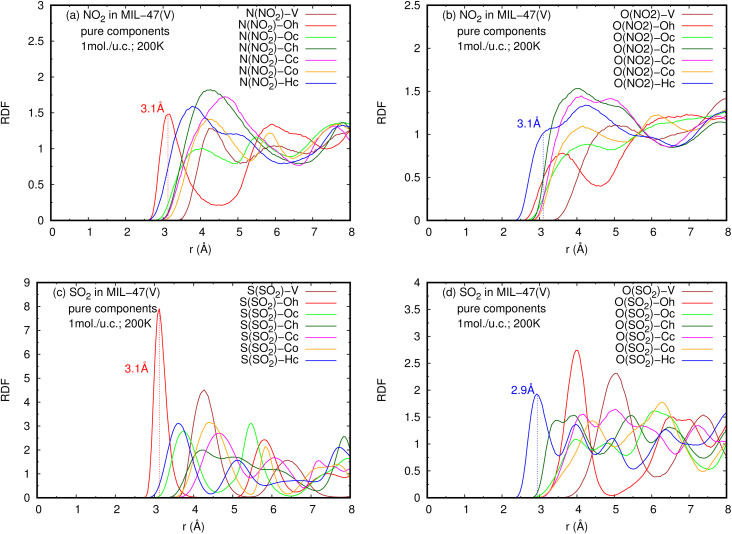
RDFS of (a), (b) NO_2_-lattice atoms and (c), (d) SO_2_-lattice atoms in pure component, at loading of 1 mol per u.c. and 200 K.

The RDFs can also provide evidence for the regional distribution of NO_2_ and SO_2_ molecules as well as their interactions with the MIL-47(V) framework. Therefore, in this study, RDFs were utilized to investigate the adsorption sites of NO_2_ and SO_2_ in the MIL-47(V). Fig. 7 shows the RDFs between atoms of NO_2_, SO_2_ and lattice atoms in the pure component, at the loading of 1 mol per u.c. The first N(NO_2_)–Oh and S(SO_2_)–Oh peaks are discovered at 3.1 Å, suggesting that Oh sites in the MIL-47(V) framework may be the preferential adsorption sites for NO_2_ and SO_2_. The peak intensities of SO_2_ are much higher than those of NO_2_, attributable to the stronger interaction of SO_2_ molecules with MIL-47(V) than that of NO_2_ molecules, which is consistent with the *D*_s_ mentioned above. Interestingly, the RDFs for the O(SO_2_)–Hc show the first peak at a distance of 2.9 Å with noticeable high intensity (see Fig. 7d), indicating a significant interaction between the O atom of SO_2_ molecules and the H atoms in the linkers of MIL-47(V), but this is not clearly seen in the RDFs for the O(NO_2_)–Hc (see Fig. 7b). Furthermore, this figure depicts that the interaction between SO_2_ and MIL-47(V) is really different from the interaction between NO_2_ and MIL-47(V).

**Fig. 8:**
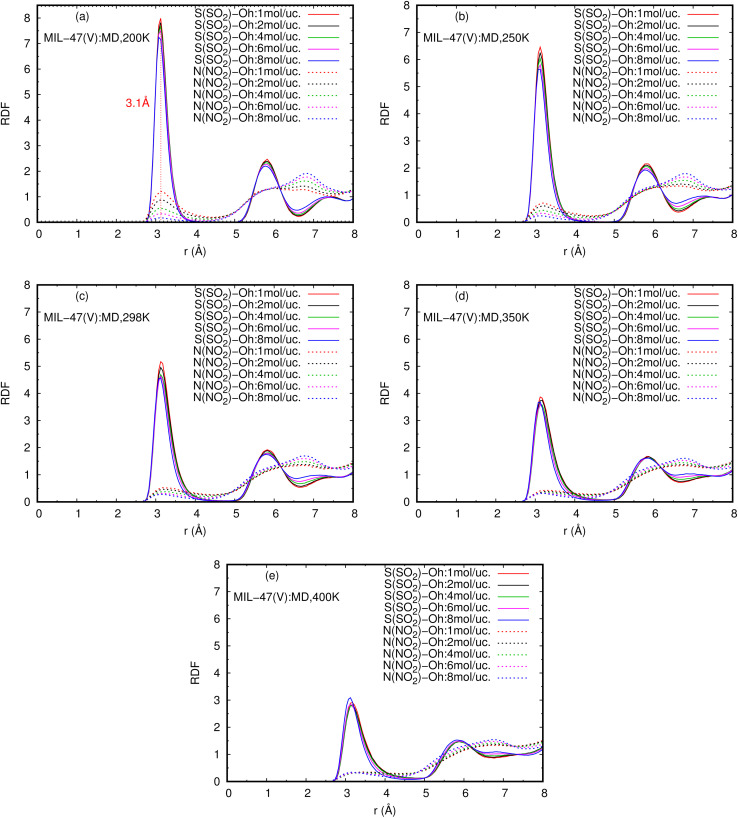
RDFS of S(SO_2_)–Oh and N(NO_2_)–Oh in mixture, at different loadings and temperatures.

Fig. 8 reports the RDFs of N(NO_2_)–Oh and S(SO_2_)–Oh in mixture, at various loadings and temperatures. The first peak of S(SO_2_)–Oh at 3.1 Å are noticeably more intense than those of NO_2_–Oh, showing that SO_2_ molecules are adsorbed at that Oh sites of MIL-47(V), while NO_2_ molecules are free to move around within the MIL-47(V) channel. This adsorption behavior is similar for both pure component and mixture (see Fig. S8 in ESI† for their RDFs in pure component).

Several experiments and simulations have recently suggested that the hydrogen bonding (HB) interaction between gas molecules (*e.g.* CO_2_, SO_2_, and H_2_O, *etc.*) and ZIFs (a subclass of MOFs) is crucial for determining the adsorption and diffusion behavior of gas molecules in MOFs.^58^ To the best of our knowledge, only a few number of research works have reported on the HB interaction of gas molecules in MILs.^63^ However, Fig. 7d shows clearly that SO_2_ can form HBs with the MIL-47(V) framework, that is, HBs between the O atoms of SO_2_ molecules and the H in the linker of MIL-47(V), but it is not the case for NO_2_.

**Fig. 9:**
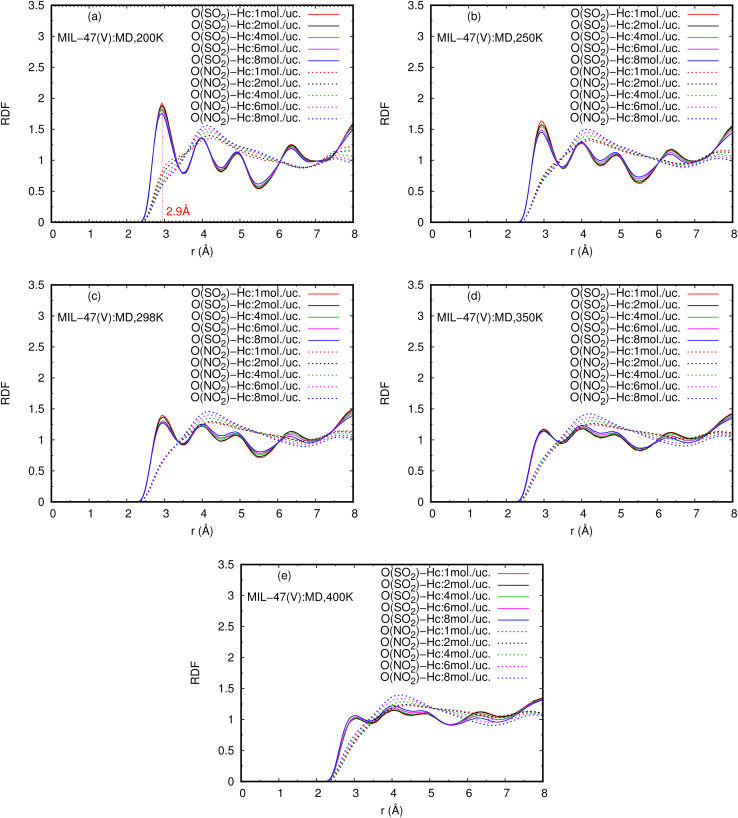
RDFS of O(SO_2_)–Hc and O(NO_2_)–Hc in mixture, at different loadings and temperatures.

Fig. 9 shows the RDFs of O(NO_2_)–Hc and O(SO_2_)–Hc in mixture, at different loadings and temperatures (see Fig. S9 in ESI† for their RDFs in pure component). The first peak of O(SO_2_)–Hc with a strong intensity is pronounced at 2.9 Å, especially at lower temperatures. This indicates the presence of HBs forming between SO_2_ molecules and MIL-47(V). However, the HB interaction decrease with increasing loading and temperature. While, it is not found for NO_2_ molecules.

#### 3.3.2 Angle distributions

The angle distribution of the O⋯H–C bond, defined as *θ*(OHC), was investigated using the MD trajectories, in order to understand the characteristics of the HB interaction between NO_2_ and SO_2_ with the MIL-47(V) framework. The vector from the H atom to its connecting C atom in MIL-47(V) linkers and the vector from the H atom to the O atom in NO_2_ and SO_2_ can be used to compute this *θ*(OHC)(OHC) angle under the condition that the O–H distance does not exceed 4 A, relating to the first peak of RDF for O–H in Fig. 9.

**Fig. 10:**
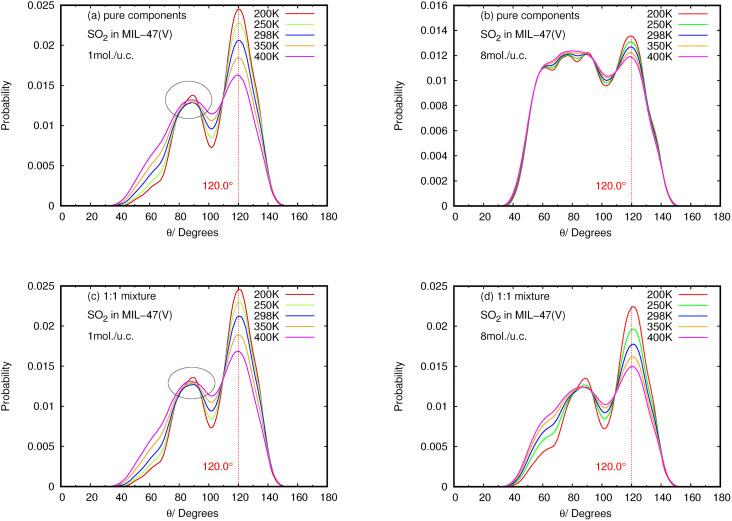
The angle distribution of O(SO_2_)–Hc–C in (a, b) pure component and (c, d) binary mixture, at different loadings and temperatures. Note that (a) and (c) are only slightly different (see the circles).

**Fig. 11:**
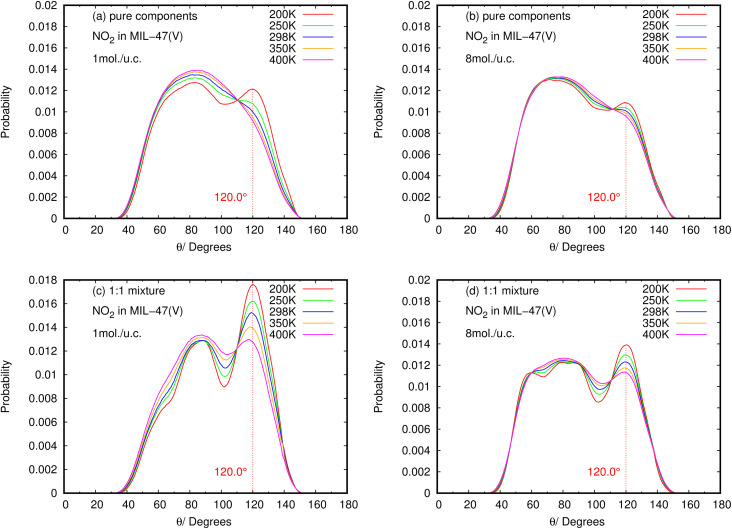
The angle distribution of O(NO_2_)–Hc–C in (a, b) pure component and (c, d) binary mixture, at different loadings and temperatures.

The results for NO_2_ and SO_2_ are presented in Fig. 10 and 11, respectively. In the case of SO_2_, the maximum value for the probability of *θ*(OHC) is 120.0°. This angle is clearly visible for both pure component and mixture, particularly at low loading and low temperature, which is well consistent with the strong interaction between SO_2_ and MIL-47(V). This suggests that 120.0° is the optimal HB binding angle for SO_2_ adsorbed in the MIL-47(V) channel. However, the probability of 120.0° decreases with increasing loading and temperature, indicating a weaker HB interaction. The probability of *θ*(OHC) in mixture is considerably higher than that in pure component, which may be related to the *E*_a_ and *D*_s_ values that were previously mentioned. In regard to CO_2_ and SO_2_ adsorption in ZIFs, the 120.0° angle of HBs was also discovered, and HB interaction is crucial for determining the adsorption and diffusion behavior of these gases in ZIFs.^58,64,65^

Contrary to SO_2_, the maximum value for the probability of *θ*(OHC) is not 120.0° in the case of pure NO_2_ component under different loading and temperature conditions. This suggests that there was no possibility of an HB interaction between NO_2_ and MIL-47(V). However, the *θ*(OHC) probability of 120.0° for NO_2_ in the mixture becomes apparent at low loading, meaning that HB interaction between NO_2_ and MIL-47(V) may occur in this circumstance, but it is much less possible than SO_2_.

#### 3.3.3 Density distributions

**Fig. 12:**
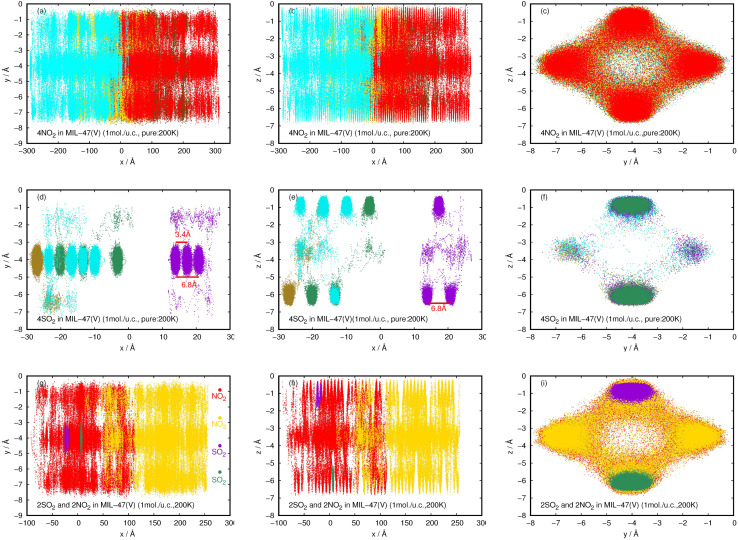
2D probability density plots of 4NO_2_ (a–c) and 4SO_2_ (d–f) for pure component, and, 2NO_2_ and 2SO_2_ for their mixture (g–i) in a MIL-47(V) channel at 200 K, displayed in *yx*, *zx*, and *zy* planes.

The investigation of MD trajectories provided the clear picture of the diffusion behavior and the precise positions of the NO_2_ and SO_2_ in the pores, at a molecular level. Fig. 12 shows the density distributions of NO_2_ and SO_2_ in the pure component and their mixture within the pores of MIL-47(V) which obtained from our MD trajectories at 200 K and a total loading of 1 mol per u.c. The pure NO_2_ molecules are freely distributed through the center of the channels along the *x*-axis, as shown in Figure12a–c, which is consistent with a 1D type diffusion process, similar to the diffusing H_2_ and CH_4_ in MIL-47(V).^45,66,67^

In contrast to pure NO_2_, the pure SO_2_ molecules behave differently in MIL-47(V), as seen in Fig. 12d–f. This dynamics study shows that a specific SO_2_ molecule stays in the Oh sites within the MIL-47(V) channel before hopping to adjacent Oh sites at a distance of 6.8 Å, which corresponds to the RDFs of S(SO_2_)–S(SO_2_), or other Oh sites on the opposite site of the channel. Hence, it is possible to divide the diffusion process of SO_2_ in MIL-47(V) into two phases. The first phase is determined by the mobility of gas molecules around the Oh sites, and the second phase involves rapid jumping to other sites. It is seen that the diffusion behavior of each gas specie are similar in both pure component and mixture (see Fig. 12g–i). Furthermore, the SO_2_ molecules exhibit similar diffusion behavior as the H_2_S molecules in MIL-47(V) reported in^45^.

**Fig. 13:**
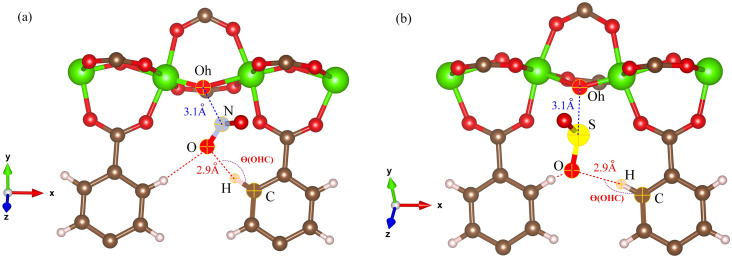
Illustration of bond distances and bond angles for (a) NO_2_ and (b) SO_2_ with the MIL-47(V) framework, obtained from MD simulations.

Fig. 13 shows the typical arrangement of a NO_2_ and SO_2_ molecule in a MIL-47(V) channel at 200 K and loading 1 mol per u.c., as well as the typical N–Oh and S–Oh distances, and *θ*(OHC) angle, which yield values consistent with those found in the RDFs discussed above. The snapshots show that NO_2_ and SO_2_ molecules are preferentially adsorbed at around the Oh sites of the framework. Simultaneously, both NO_2_ and SO_2_ molecules attempt to link their N or S atoms to the Oh atom, while their O atoms connect to the H atom in the framework's linker.

### 3.4 Diffusion selectivity (*S*_diff_)

**Fig. 14:**
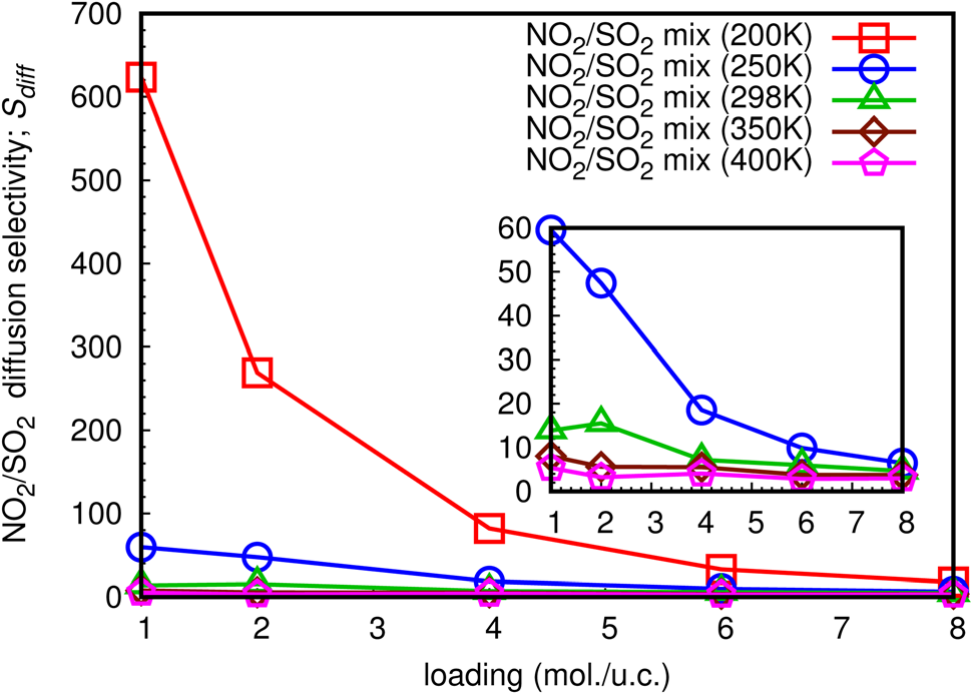
The loading dependence of *S*_diff_ of NO_2_ and SO_2_ in mixture within the MIL-47(V) framework.

The diffusion selectivity of gases in MOFs depends on several factors, including the size of the gas molecules, the size and shape of the pores in the MOF, and the chemical interactions between the gas molecules and the MOF framework. Additionally, the ability to selectively transport gases through MOFs has many potential applications in areas, such as gas storage, separation, and purification, as well as in catalysis and sensing. In this work, diffusion selectivity for a NO_2_/SO_2_ binary mixture is defined as *S*_diff_ = *D*_s,NO_2__/*D*_s,SO_2__, where *D*_s,NO_2__ is *D*_s_ of NO_2_, and *D*_s,SO_2__ is *D*_s_ of SO_2_. The loading dependence for *S*_diff_ at different temperatures is shown in Fig. 14. For all studied temperatures, the *S*_diff_ exhibits the same pattern, with the a maximum at low total loading of 1 mol per u.c. and decreasing as the loading increases. At total loading 1 mol per u.c. of 200 K, the extraordinarily high *S*_diff_ of 623.4 is obtained, indicating that NO_2_ can diffuse through the MIL-47(V) lattice significantly faster than SO_2_ in their mixture, especially at low loading. The sub-figure shows that with a total loading of 1 mol per u.c. of 250 K, *S*_diff_ is likewise high of 59.5 and follows the same trend as at 200 K. Furthermore, for temperatures equal to or greater than 298 K, it is not greatly impacted by temperature and loading, with maximum values of 15.5, 8.1, and 5.4 found for 298 K, 350 K, and 400 K, respectively.

## 4 Conclusion

In this work, the classical MD simulations have been performed to investigate the *D*_s_ of pure NO_2_, SO_2_, and their equimolar binary mixture in the MIL-47(V) at different loadings and temperatures. Our MD simulations shows that NO_2_ molecules exhibit different diffusion behavior from SO_2_ in both pure component and binary mixture in MIL-47(V). The *D*_s_ of NO_2_ molecules in MIL-47(V) are approximately two orders of magnitude greater than those of SO_2_ at low loadings and temperatures, in the cases of both pure component and binary mixture. The diffusion of NO_2_ is affected by the steric hindrance between diffusion molecules at higher loading, where its *D*_s_ value decreases monotonically with loading indicating Type I self-diffusion. While, *D*_s_ of SO_2_ are more sensitive to loading and temperature than those of NO_2_ and show two types of diffusion mechanism. The SO_2_ molecules in MIL-47(V) exhibit type I self-diffusion at temperature higher than 298 K, showing that its *D*_s_ decreases with increasing loading, similar to NO_2_. At temperatures equal to or less than 298 K, the *D*_s_ of SO_2_ indicates Type IV self-diffusion, where its *D*_s_ increases with increasing loading, reaches a maximum and then decreases with increasing loading. Type IV self-diffusion is mostly due to the specific interactions between the lattice and the gas molecules, and the maximum in *D*_s_ is determined by the number of adsorption sites.

The obtained *E*_a_ of SO_2_ in MIL-47(V) in mixture is larger than in pure component, indicates that SO_2_ molecules have slower diffusion in mixture than in pure component. The maximum *E*_a_ of SO_2_ are 16.43 and 18.35 kJ mol^−1^ for the pure component and the mixture, respectively. In case of NO_2_, their *E*_a_ in mixture are only slightly depend on the loading and also slightly lower than those in pure component. The averaged *E*_a_ value of NO_2_ for the pure component and the mixture are only 2.69 and 1.89 kJ mol^−1^, respectively. The RDFs of N(NO_2_)–Oh and S(SO_2_)–Oh suggested that Oh in MIL-47(V) could be the preferential adsorption sites for NO_2_ and SO_2_, where the peak intensities of SO_2_ are much higher than those of NO_2_, relating to their *D*_s_. In addition, the higher intensity of S(SO_2_)–Oh displays that SO_2_ molecules are adsorbed at the Oh site, while NO_2_ can move freely within the lattice. The RDFs of O(SO_2_)–Hc with high intensity is found to indicate the HB between the O atoms of SO_2_ and the H atoms of the organic linkers of MIL-47(V), but it could not occur for NO_2_.

Furthermore, the calculated angle distributions of the O⋯H–C bond, so-called *θ*(OHC), show the optimal HB binding angle of 120° for SO_2_ adsorbed in the MIL-47(V) channel, and the probability of 120° is higher at low loading and temperatures. The probability of *θ*(OHC) for NO_2_ suggested that there is no HB interaction between NO_2_ and MIL-47(V) in pure component, nevertheless it may occur in mixture, but it is much less possible than SO_2_.

The probability densities illustrate that NO_2_ are freely distributed through the center of the channel along the *x*-axis, showing to the 1D diffusion. While, SO_2_ molecules are found the stay close to the Oh sites in the MIL-47(V) and it shows their jump diffusion between the Oh sites.

The *S*_diff_ for a NO_2_/SO_2_ binary mixture exhibit the loading dependence at different temperature, where a maximum value is found at loading of 1 mol per u.c., and it decreases with increasing loading. Interestingly, the extraordinarily high *S*_diff_ of 623.4 is detected at a total loading of 1 mol per u.c. and temperature of 200 K, meaning that NO_2_ can diffuse through the MIL-47(V) lattice significantly faster than SO_2_ in their mixture, especially at low loading. Finally, our simulations in this work provide a clear picture at molecular level of the adsorption and diffusion mechanism of NO_2_ and SO_2_ in the MIL-47(V) material. This is valuable information for developing new MOF materials for gas adsorption and separation.

## Data availability

The authors confirm that the data supporting the findings of this study are available within the article and its ESI.†

## Conflicts of interest

There are no conflicts to declare.

## Supplementary Material

RA-013-D3RA02724D-s001
