# Supplementary material for: Molecular adsorption and self-diffusion of NO2, SO2, and their binary mixture in MIL-47(V) material
Source: RSC Adv. 2023 Jun 23;13(28):19207–19. doi: 10.1039/d3ra02724d (PMC10289206; doi:10.1039/d3ra02724d)
Supplement: RA-013-D3RA02724D-s001 [file RA-013-D3RA02724D-s001.pdf]

Electronic Supplementary Material (ESI) for RSC Advances.
This journal is © The Royal Society of Chemistry 2023

Supplementary Material

for

Molecular Adsorption and Self-Diffusion of NO_2, SO_2, and Their Binary Mixture in MIL-47(V) Material

Kompichit Seehamart[1], Wutthikrai Busayaporn[2] and Rungroj Chanajaree[3,*]

[1]*Department of Applied Physics, Faculty of Engineering, Rajamangala University of Technology Isan, Khon Kaen Campus, Khon Kaen 40000, Thailand.*

[2]*Synchrotron Light Research Institute (Public Organization), Nakhon Ratchasima 30000, Thailand.*

[3]*Metallurgy and Materials Science Research Institute (MMRI), Chulalongkorn University, Bangkok 10330, Thailand.*

[*]corresponding author, email: Rungroj.Ch@chula.ac.th

1. The MD simulation boxes of SO_2 and NO_2 molecules in mixture within the MIL-47(V) frameworks

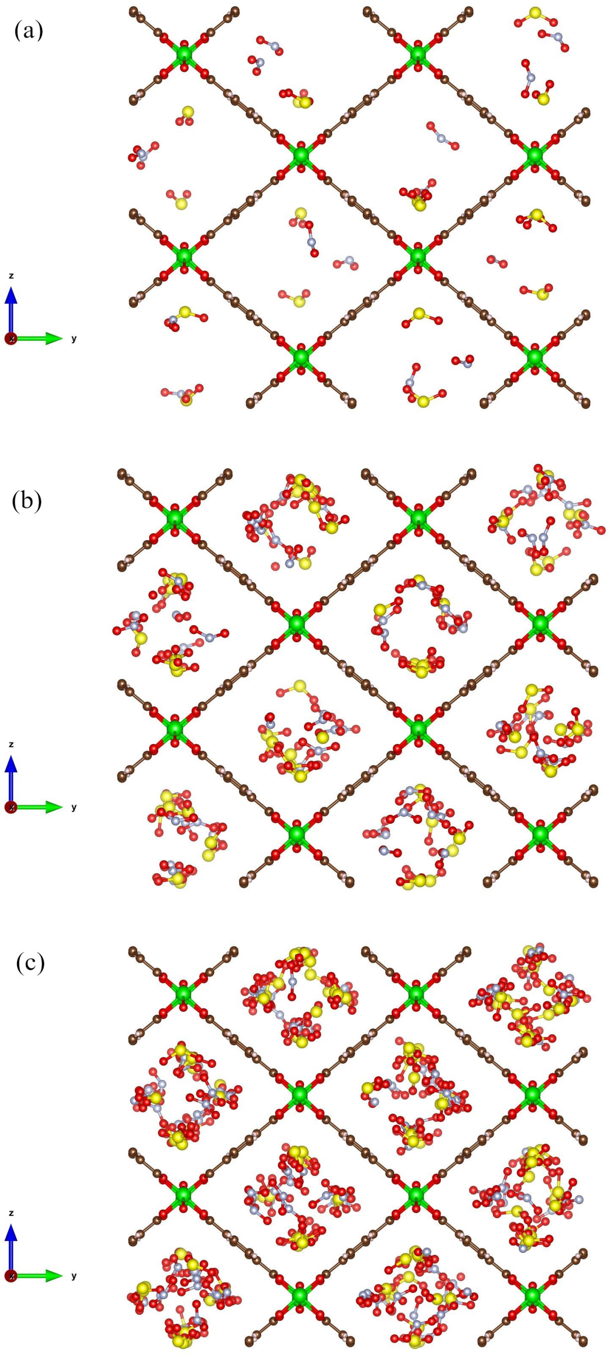

Figure S1: MD simulation boxes of NO_2 and SO_2 in mixture within MIL-47(V) at temperature of 298 K, and at loadings of (a) 1, (b) 4, and (c) 8 mol./u.c.

2. Mean Square Displacements (MSDs) of SO_2 and NO_2 within 1D channel of MIL-47(V)

The MSDs of SO_2 and NO_2 molecules in pure component and in mixture within 1D channel of MIL-47(V) framework at temperatures of 200-450 K at loading of 1 mol./u.c. are shown in Figures S2 and S3. They show linear relation which indicates good statistics and normal diffusion occurs at the time scale. The log-log plot of MSDs of SO_2 and NO_2 are illustrated in Figure S4. It indicates the sub-diffusion of SO_2 in MIL-47(V).

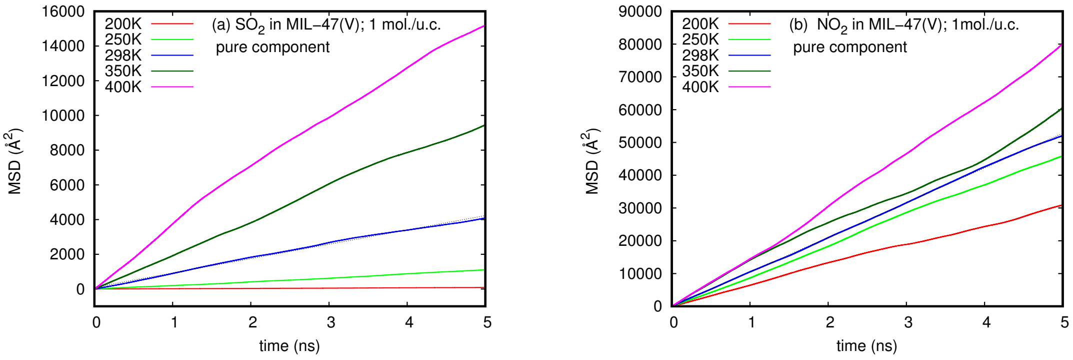

Figure S2: MSDs of SO_2 and NO_2 molecules in pure component within 1D channel of MIL-47(V). The fit to the long-time behavior is shown by the dashed line.

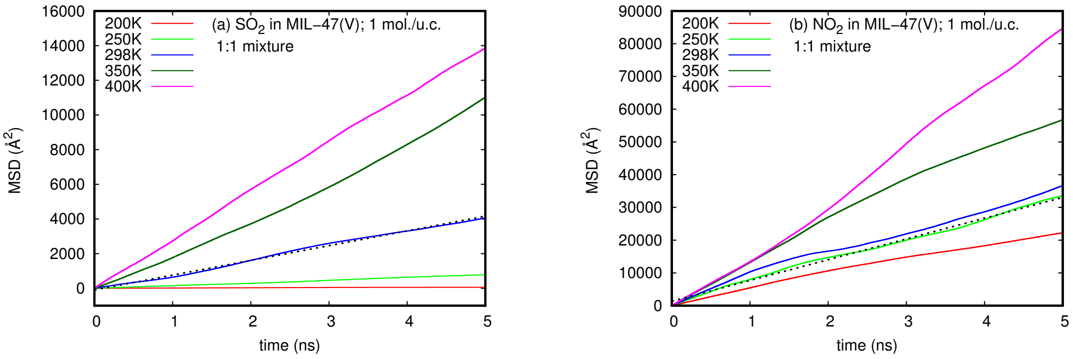

Figure S3: MSDs of SO_2 and NO_2 molecules in mixture within 1D channel of MIL-47(V). The fit to the long-time behavior is shown by the dashed line.

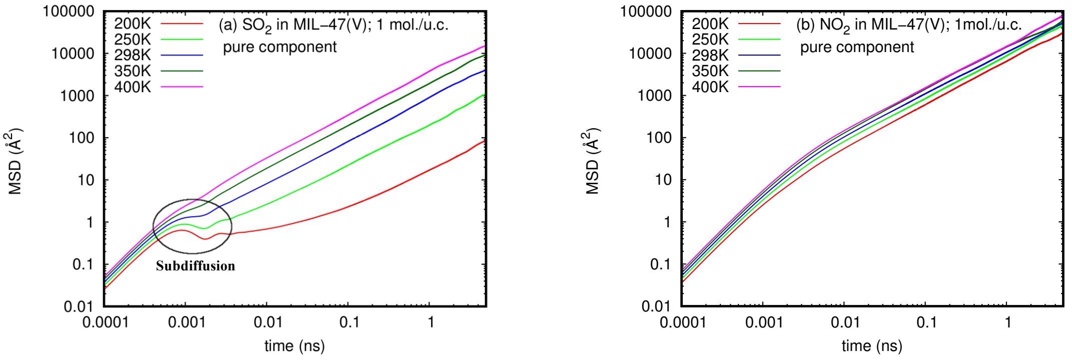

Figure S4: Log-log plot of the MSDs of SO_2 and NO_2 molecules in pure component within 1D channel of MIL-47(V).

3. Mean Square Displacements (MSDs) of SO_2 and NO_2 within MIL-47(V) in x-, y- and z-axes

The MSDs of SO_2 and NO_2 molecules in pure component within MIL-47(V) framework at temperatures of 298 K at loading of 1 mol./u.c. They show that the diffusion occurs only in x-axis.

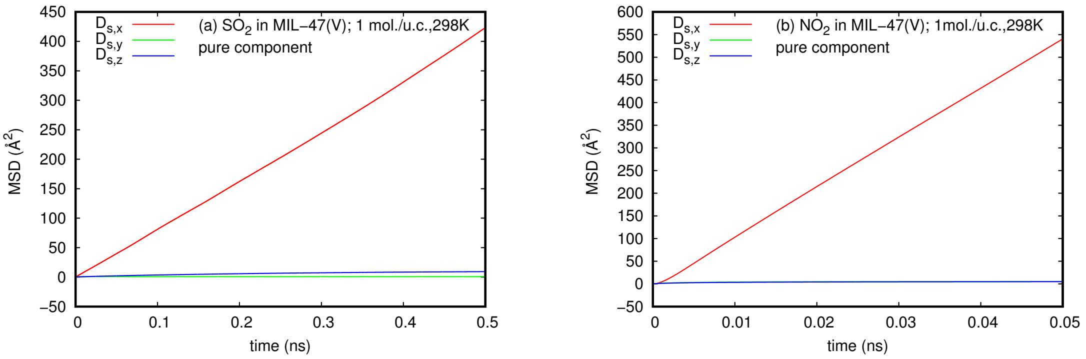

Figure S5: MSDs of SO_2 and NO_2 molecules within MIL-47(V) in x-, y- and z-axes

4. The relationship between ln(D_s) and 1/T for SO_2 and NO_2 in pure component within MIL-47(V) at different loadings.

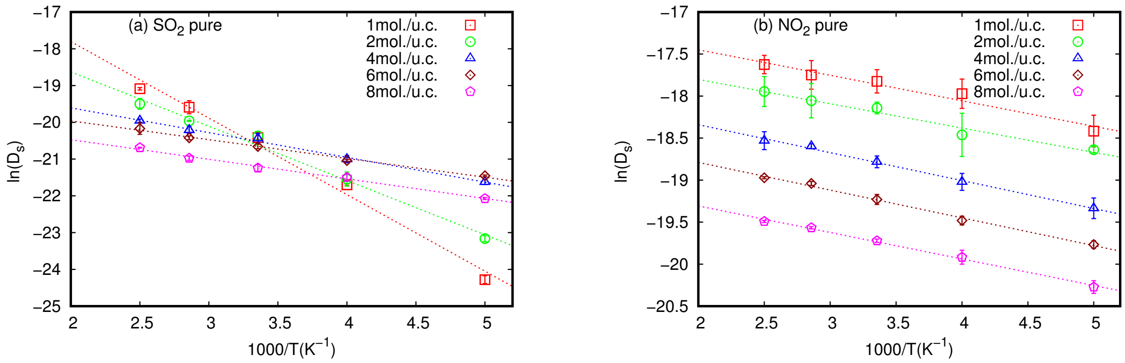

Figure S6: Inverse temperature dependence of the simulated D_s of (a) SO_2 and (b) NO_2 in pure component within MIL-47(V) at different loadings. The dashed line represent the linear fit for an Arrhenius relationship.

5. Radial Distribution Functions (RDFs)

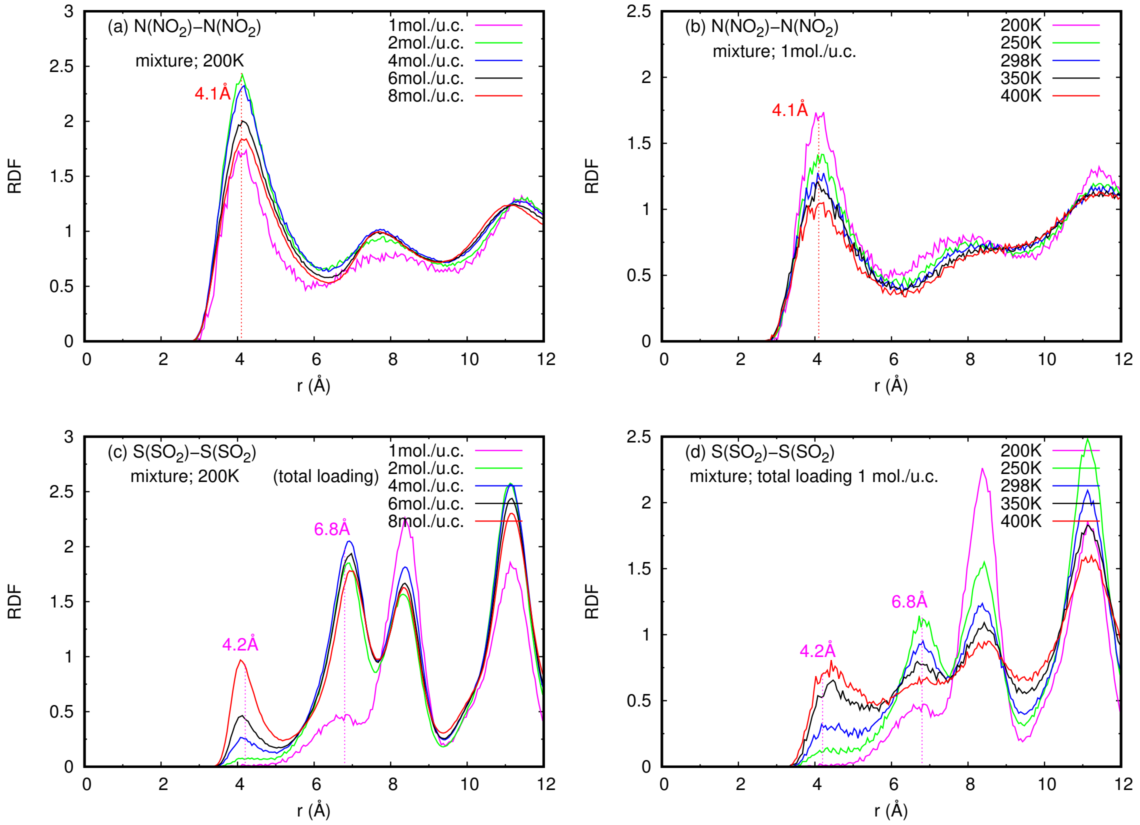

Figure S7: RDFS of (a)-(b) NO_2-NO_2 and (c)-(d) SO_2-SO_2 in mixture, at different loadings and temperatures.

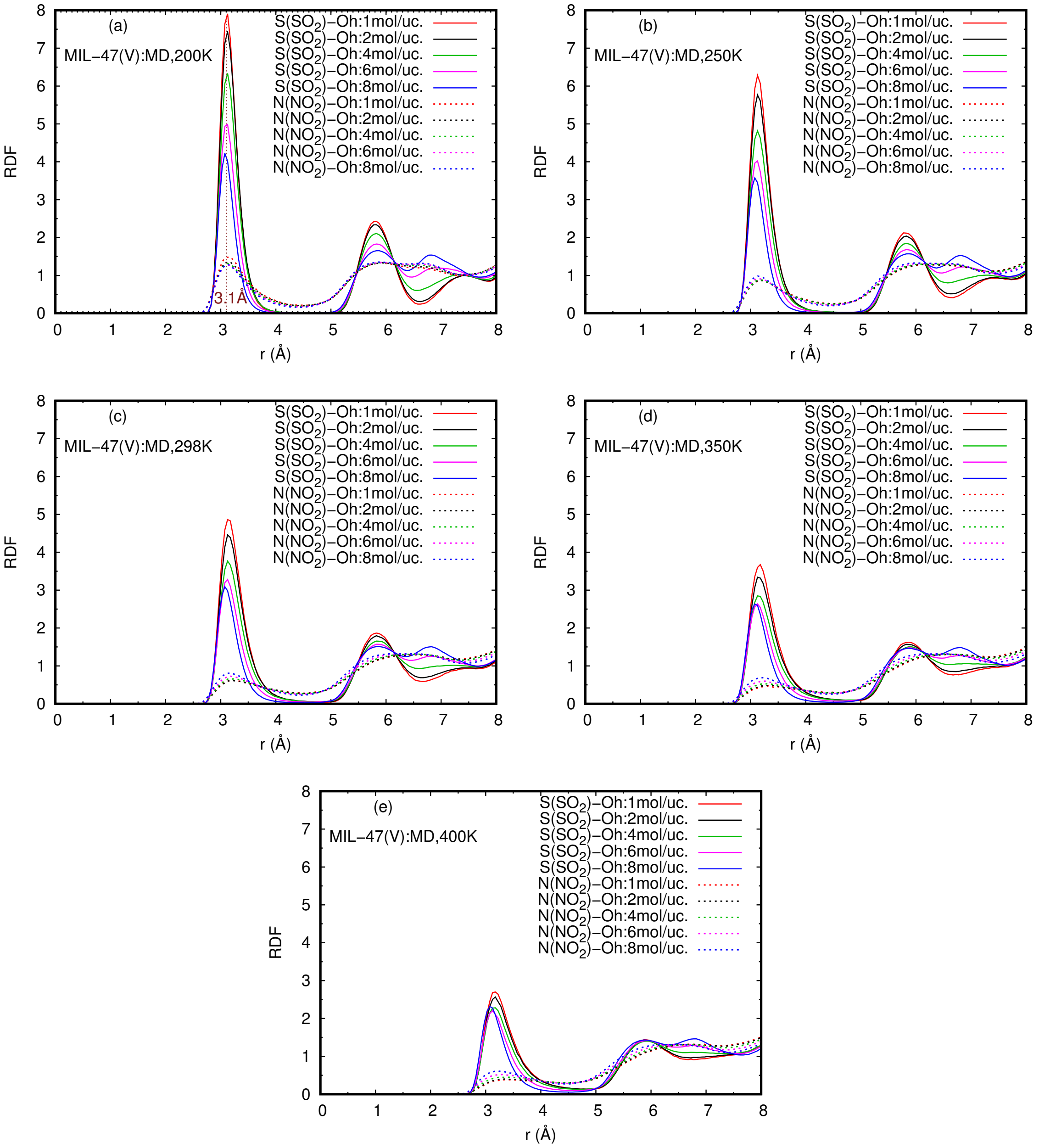

Figure S8: RDFS of S(SO_2)-Oh and N(NO_2)-Oh in pure component, at different loadings and temperatures.

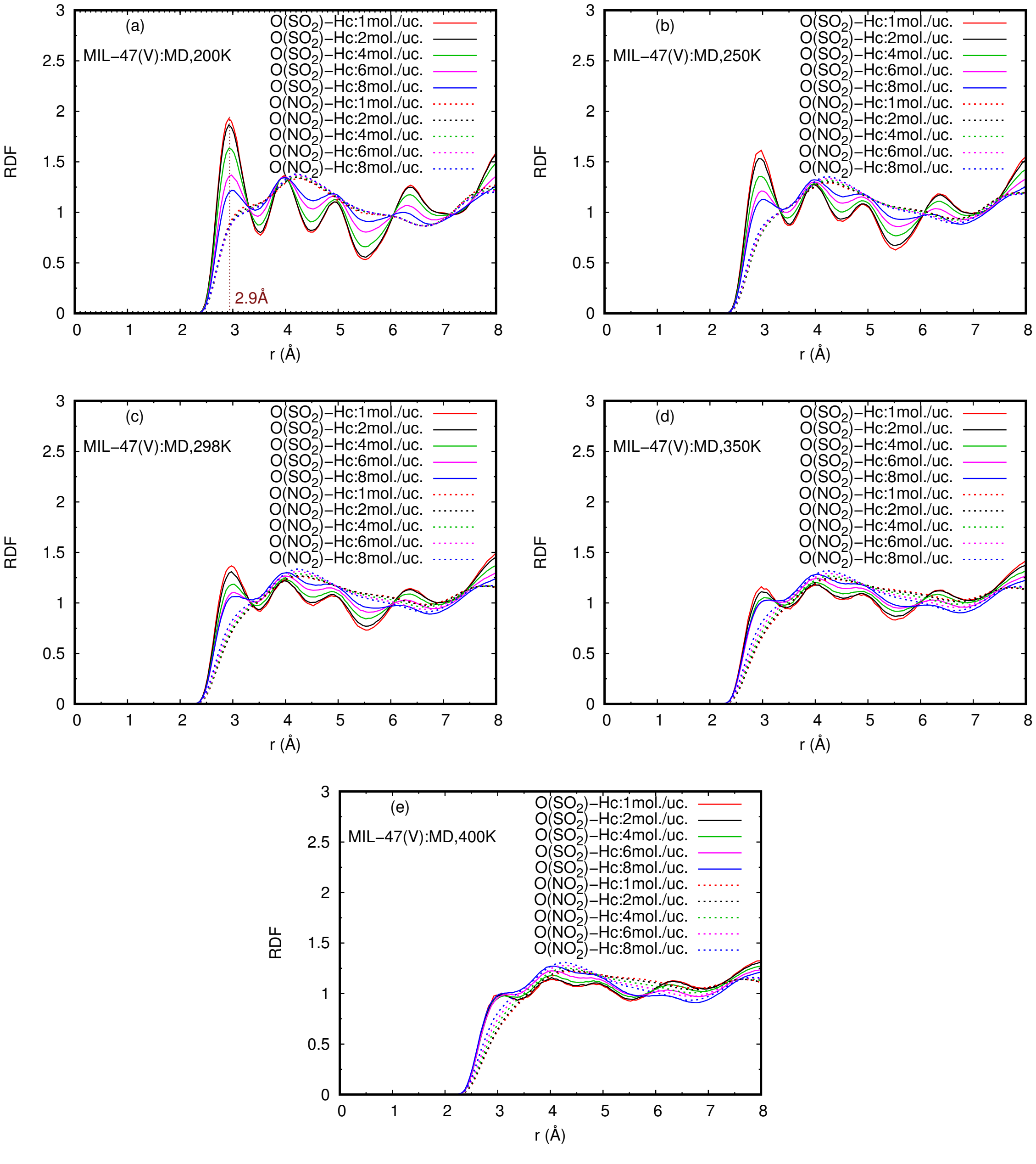

Figure S9: RDFS of O(SO_2)-Hc and O(NO_2)-Hc in pure component, at different loadings and temperatures.

Table S1: Self-diffusion coefficients (D_s) of NO_2 and SO_2 in pure component ($m^2{\cdot}s^{-1}$).

Total loading	200 K		250 K		298 K		350 K		400 K	
(mol./u.c.)	NO_2	SO_2	NO_2	SO_2	NO_2	SO_2	NO_2	SO_2	NO_2	SO_2
1	1.00×10^{-8}	2.86×10^{-11}	1.57×10^{-8}	3.77×10^{-10}	1.81×10^{-8}	1.36×10^{-9}	1.96×10^{-8}	3.11×10^{-9}	2.22×10^{-8}	5.13×10^{-9}
2	8.04×10^{-9}	8.73×10^{-11}	9.60×10^{-9}	4.40×10^{-10}	1.32×10^{-8}	1.43×10^{-9}	1.44×10^{-8}	2.14×10^{-9}	1.61×10^{-8}	3.40×10^{-9}
3	5.39×10^{-9}	1.74×10^{-10}	6.99×10^{-9}	6.58×10^{-10}	8.60×10^{-9}	1.33×10^{-9}	1.04×10^{-8}	1.97×10^{-9}	1.12×10^{-8}	2.81×10^{-9}
4	4.00×10^{-9}	4.08×10^{-10}	5.49×10^{-9}	7.56×10^{-10}	6.96×10^{-9}	1.33×10^{-9}	8.38×10^{-9}	1.67×10^{-9}	8.96×10^{-9}	2.16×10^{-9}
5	3.28×10^{-9}	5.47×10^{-10}	4.60×10^{-9}	7.62×10^{-10}	5.28×10^{-9}	1.26×10^{-9}	6.09×10^{-9}	1.68×10^{-9}	7.42×10^{-9}	2.06×10^{-9}
6	2.60×10^{-9}	4.82×10^{-10}	3.46×10^{-9}	7.28×10^{-10}	4.45×10^{-9}	1.07×10^{-9}	5.38×10^{-9}	1.35×10^{-9}	5.76×10^{-9}	1.74×10^{-9}
7	2.56×10^{-9}	3.94×10^{-10}	2.87×10^{-9}	5.84×10^{-10}	3.55×10^{-9}	8.14×10^{-10}	4.17×10^{-9}	1.07×10^{-9}	4.62×10^{-9}	1.36×10^{-9}
8	1.57×10^{-9}	2.60×10^{-10}	2.24×10^{-9}	4.60×10^{-10}	2.72×10^{-9}	5.96×10^{-10}	3.18×10^{-9}	7.79×10^{-10}	3.43×10^{-9}	1.04×10^{-9}

Table S2: Self-diffusion coefficients (D_s) of NO_2 and SO_2 in mixture ($m^2{\cdot}s^{-1}$).

Total loading	200 K		250 K		298 K		350 K		400 K	
(mol./u.c.)	NO_2	SO_2	NO_2	SO_2	NO_2	SO_2	NO_2	SO_2	NO_2	SO_2
1	1.09×10^{-8}	1.74×10^{-11}	1.81×10^{-8}	3.05×10^{-10}	1.53×10^{-8}	1.10×10^{-9}	2.20×10^{-8}	2.73×10^{-9}	2.27×10^{-8}	4.25×10^{-9}
2	7.80×10^{-9}	2.90×10^{-11}	1.12×10^{-8}	2.37×10^{-10}	1.18×10^{-8}	7.59×10^{-10}	1.22×10^{-8}	2.17×10^{-9}	1.47×10^{-8}	4.55×10^{-9}
4	4.37×10^{-9}	5.54×10^{-11}	4.94×10^{-9}	2.66×10^{-10}	5.99×10^{-9}	8.31×10^{-10}	7.39×10^{-9}	1.34×10^{-9}	7.89×10^{-9}	1.94×10^{-9}
6	2.45×10^{-9}	7.46×10^{-11}	3.34×10^{-9}	3.16×10^{-10}	3.62×10^{-9}	6.06×10^{-10}	4.17×10^{-9}	1.10×10^{-9}	4.33×10^{-9}	1.52×10^{-9}
8	1.60×10^{-9}	9.05×10^{-11}	1.77×10^{-9}	2.74×10^{-10}	2.00×10^{-9}	4.32×10^{-10}	2.38×10^{-9}	6.31×10^{-10}	2.69×10^{-9}	9.01×10^{-10}
